# Hydrogen sulfide treatment protects against renal ischemia-reperfusion injury via induction of heat shock proteins in rats

**DOI:** 10.22038/ijbms.2018.29706.7170

**Published:** 2019-01

**Authors:** Yang Du, Xiu-heng Liu, Heng-cheng Zhu, Lei Wang, Zhi-shun Wang, Jin-zhuo Ning, Cheng-cheng Xiao

**Affiliations:** 1 Department of Urology, Renmin Hospital of Wuhan University, Hubei, China; 2 Physician, Department of Urology, Renmin Hospital of Wuhan University, Hubei, China

**Keywords:** Hydrogen sulfide, Heat shock protein 70, Heat shock protein 27, Heme oxygenase 1, Ischemia-reperfusion injury, Rat, Renal

## Abstract

**Objective(s):**

Hydrogen sulfide (H_2_S) attenuates ischemia-reperfusion injury (IRI) in different organs. However, its mechanism of action in renal IRI remains unclear. The present study investigated the hypothesis that H_2_S attenuates renal IRI via the induction of heat shock proteins (HSPs).

**Materials and Methods:**

Adult Wistar rats were subjected to unilateral renal ischemia for 45 min followed by reperfusion for 6 hr. One group of rats underwent I/R without treatment, one group was administered 150 μmol/l sodium hydrosulfide (NaHS) prior to I/R, one group was injected with 100 mg/kg quercetin (an HSP inhibitor) intraperitoneally prior to I/R, and another group received quercetin prior to I/R and treatment with NaHS following I/R. Two other groups underwent a sham operation and one of them received 150 μmol/l NaHS following the sham operation whereas the other received no treatment. Renal function and histological changes were compared and relevant indices of oxidative stress, apoptosis, and inflammation were examined.

**Results:**

IRI increased serum creatinine and blood urea nitrogen concentrations, promoted lipid peroxidation by elevating malondialdehyde levels, suppressed superoxide dismutase activity, stimulated inflammation by inducing NF-kB, IL-2, and TLR-4 expression, and increased renal apoptosis. Levels of HSP 70, heme-oxygenase-1 (HO-1) and HSP 27 were increased following IRI and reversed following H_2_S treatment. H_2_S attenuated changes observed in pathology, lipid peroxidation, inflammation, and apoptosis following IRI. The administration of quercetin reversed all protective effects of H_2_S.

**Conclusion:**

The present study indicated that H_2_S protected renal tissue against IRI induced lipid peroxidation, inflammation, and apoptosis, which may be attributed to the upregulation of HSP 70, HO-1, and HSP 27.

## Introduction

Renal ischemia-reperfusion injury (IRI) is a pathological process that usually occurs in transplantation, partial nephrectomy, shock, and other renal thrombotic angiopathy diseases (1). Kidneys are prone to IR injury, the main cause can be ascribed to its high perfusion level. Renal IRI can lead to acute renal failure, delayed graft function, graft rejection and increase early mortality in patients subjected to kidney transplantation (2). According to a previous study, the mortality of acute renal failure (ARF) associated with renal IR injury is about 30%–50% (3), and nearly 10% of renal allografts fail additionally in the first year after transplantation, moreover, the average risk of renal failure increases about 3%–5% (4). In the past few years, hydrogen sulfide (H_2_S) as a novel gasotransmitter has been demonstrated to possess a wide range of pharmaceutical effects, including vasodilation, anti-oxidative stress, anti-inflammatory, and anti-apoptosis effects (5). Due to such mentioned effects, H_2_S has also been shown to protect against IR injury in many organs. Though we have known such protective effect of H_2_S, the mechanism through which the gas exerts its protective properties is still not fully understood.

Heat shock proteins (HSPs) are a group of stress-responsive proteins expressed widely in various organs. These proteins are categorized into different subfamilies according to their molecular weight. Previous studies have demonstrated that HSPs are involved in multiple regulations of the cell signaling pathway (6). Zhang *et al.* reported that the expression levels of HSPs are highly sensitive to IR injury in rat kidney, and three of those including HSP 70, HO-1, and HSP 27 increased more obviously (7), similar results also have been reported in Guo *et. al.* study (8). HSP 70 is a chaperone protein that plays a key role in the regulation of apoptotic signaling (9). HO-1 has been studied widely for its antioxidant and anti-inflammatory functions, as well as its abilities of maintaining microcirculation and suppressing immunological rejection (10). HSP 27, as a member of the small molecular family of HSPs, is also vitally important in inhibiting cellular apoptosis, keeping intracellular homeostasis of redox, and maintaining mitochondrial integrity (11).

 Therefore, we hypothesize that H_2_S may protect against renal IR injury by induction of HSPs. To the best of our knowledge, the potential that HSP induced by H_2_S in kidney and the further influence against renal IR have not been illustrated. So in the present study, we verified whether H_2_S induced higher expression of HSP 70, HO-1, and HSP 27, thereby ameliorating oxidative stress, inflammatory responses, and cellular apoptosis, so as to attenuate renal IR injury of rats.

## Materials and Methods

***Animals***

Male Wistar rats, weighing 250–300 g, aged 6–8 weeks, were obtained from the Center of Experimental Animals in Wuhan University Medicine College (Hubei, China). All rats were housed in a standard environment with 12-hr day-night cycles, free access to water and standard laboratory diet. All experimental procedures are approved by the Institutional Animal Care and Use Committee of Wuhan University, and the Guide for the Care and Use of Laboratory Animals (1996) was followed.

***Experimental protocol***

A total of 48 rats were randomly divided into 6 groups (n=8, each): sham group, sham + sodium hydrosulfide (NaHS) group, IR group, IR + quercetin (HSP inhibitor) group, NaHS group, and NaHS + quercetin group. All rats were anesthetized with ketamine (30 mg/kg), maintained with 1% isoflurane, and placed on a homoeothermic table to keep a body temperature of 37 ^°^C during surgery. Briefly, a right nephrectomy was performed through a midline abdominal incision in order to remove confounding protective effects of the contralateral functioning kidney. In IR, IR + quercetin, NaHS, and NaHS + quercetin groups, the left renal pedicle was subsequently occluded by atraumatic clamping for 45 min, then followed by 6 hr of reperfusion. Sham group and Sham + NaHS group were only subjected to a right nephrectomy. 

***Intervention study***

Sodium hydrosulfide monohydrate (CAS No: 140650-84-6, purity >90%) was purchased from Sigma-Aldrich Co LLC. It was diluted to form a 150 umol/l solution with 1 mol/l phosphate buffer saline (PBS). During occlusion, the abdomen was injected with 10 ml of PBS (IR, Sham, and IR + quercetin groups) or 150 µmol/l NaHS (NaHS, Sham + NaHS, and NaHS + quercetin groups) and the incision was sutured to minimize. After 6 hr of reperfusion, all rats were sacrificed with an overdose of pentobarbital sodium. At the time of sacrifice, the left kidney was removed under fully maintained anesthesia. After removal, tissues were fixed in 4% paraformaldehyde or immediately frozen and stored at -80 ^°^C for subsequent analysis.

***Serum assays***

After 6 hr of reperfusion, 3 ml blood samples extracted from cardiac blood were reserved and analyzed. The absorbance was measured via a spectrophotometer (UV-1700; Shimadzu Corporation, Tokyo, Japan), after that, the concentrations of BUN and creatinine were calculated.

***Malondialdehyde (MDA) and superoxide dismutase (SOD) assays***

The frozen renal tissues were homogenized and centrifuged, then, the supernatants were collected. The MDA level and SOD activity were measured spectrophotometrically by using MDA and SOD assay kits (Nanjing Jiancheng Bioengineering Institute, China). The MDA level was detected according to the absorbance at 532 nm and expressed as nmol/mg protein. The SOD activity was expressed as U/mg protein.

***Histological evaluation***

After fixing the renal tissues in 4% paraformaldehyde, they were embedded in paraffin and then incised with an average thickness of 4 μm. The sections were stained with hematoxylin and eosin (H&E) after deparaffinage and hydration. Cellular apoptosis in kidneys was detected by Terminal deoxynucleotidyl-transferase-mediated dUTP nick-end labeling (TUNEL) assay. All morphologic assessments were given by an experienced renal pathologist in a blinded fashion.

**Table I:** Primer sequences used in quantitative polymerase chain reaction

Name	Primer	Sequence 5’ to 3’
IL-2	Forward	CACTGACGCTTGTCCTCCTTGT
	Reverse	GTTTCAATTCTGTGGCCTGCT
TLR-4	Forward	TCCACAAGAGCCGGAAAGTT
	Reverse	TGAAGATGATGCCAGAGCGG
caspase-3	Forward	GACAACAACGAAACCTCCG
	Reverse	AGGGTTAGCTGCATCGACA
GAPDH	Forward	ACAGCAACAGGGTGGTGGAC
	Reverse	TTTGAGGGTGCAGCGAACTT

**Figure 1:**
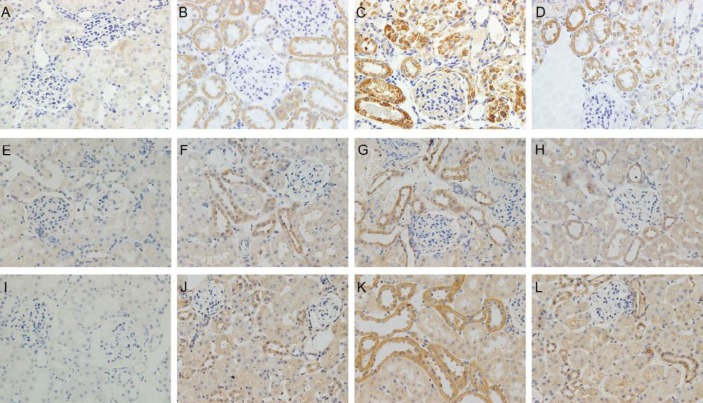
(A-D) Expression of HSP70 in rat kidneys following 6 hr of reperfusion (magnification, ×400). (E-H) Expression of HO-1 in rat kidneys following 6 hr of reperfusion. (I-L) Expression of HSP27 in rat kidneys following 6 hr of reperfusion. (A, E, I) Sham group: Sections from a sham-operated rat. (B,F,J) I/R group: Sections from rat subjected to I/R treatment. (C,G,K) NaHS group: Sections from rat subjected to I/R and NaHS treatment. (D, H, L) Quercetin + NaHS group: Sections from rat subjected to I/R and NaHS treatment after the administration of quercetin. HSP, heat shock protein; I/R, ischemia and reperfusion; NaHS, sodium hydrosulfide

**Figure 2:**
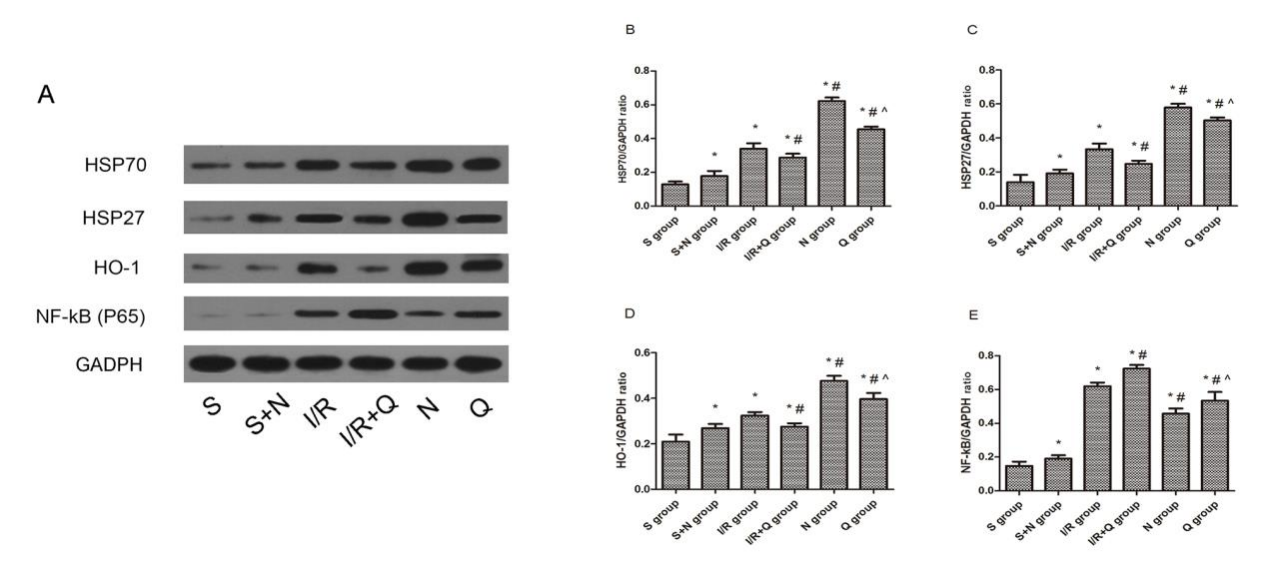
Representative Western blots showed the effects of H2S on HSP70, HSP27, HO-1, and NF-kB (p65) expression in rat kidneys after 45 min of ischemia followed by 24 hr of reperfusion. GADPH was used to show equal amounts of protein loading in each lane. (A) Representative Western blots showed the effects of H2S on HSP70, HSP27, HO-1, and NF-kB (p65) expression. Relative band densities of (B) HSP70, (C) HSP27, (D) HO-1, and (E) NF-kB (p65) to the mean value of the control. Bars represent means ± standard error of the mean (n=8 per group); *P<0.05 vs the sham group, #*P*<0.05 vs the I/R group, ^*P*<0.05 vs the NaHS group. S, sham group; S+N, sham + NaHS group; I/R, ischemia-reperfusion group; I/R+Q group, ischemia-reperfusion +quercetin group; N, NaHS (sodium hydrosulfide) group; Q, quercetin.+ NaHS group

**Figure 3:**
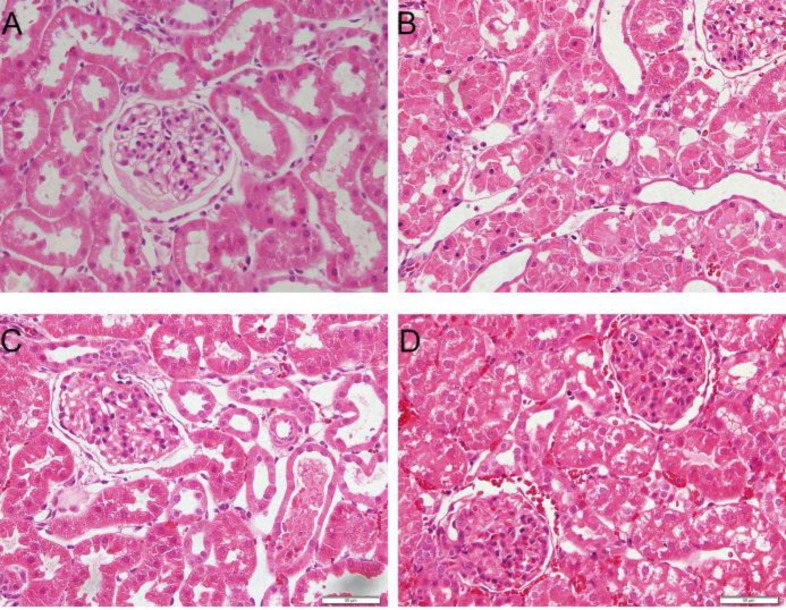
Microphotographs of kidney tissues in four groups at 6 hr post-reperfusion (hematoxylin and eosin stain; magnification, ×400). (A) Sham group showed no obvious morphological changes. (B) The ischemia-reperfusion group showed severe pathological and morphological changes, tubular dilatation, and cellular edema, with partly visible necrosis and tubular cells. (C) the NaHS group showed an attenuation of the pathological changes. (D) Quercetin + NaHS suppressed the renoprotective effects of NaHS. NaHS, sodium hydrosulfide

**Figure 4:**
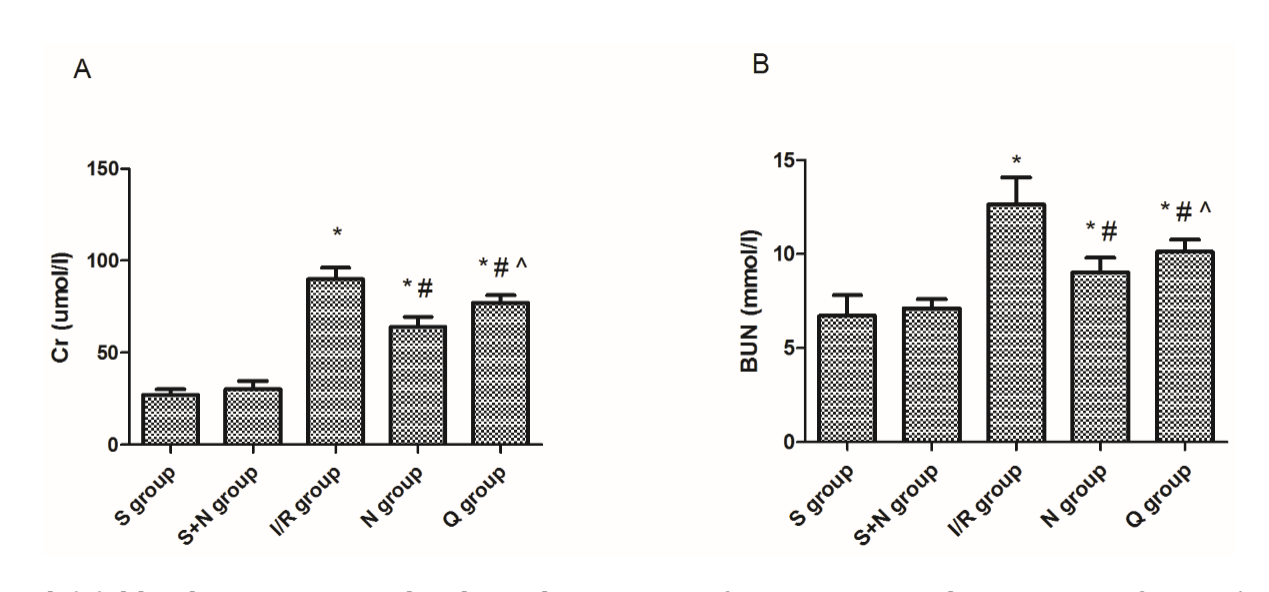
(A) Creatinine and (B) blood urea nitrogen levels in the serum in five groups at 6 hr post-reperfusion. *P<0.05 vs the sham group, #*P*<0.05 vs the I/R group, ^*P*<0.05 vs the NaHS group. S, sham group; S+N, sham + NaHS group; I/R, ischemia-reperfusion group; N, NaHS (sodium hydrosulfide) group; Q, quercetin + NaHS group

**Figure 5:**
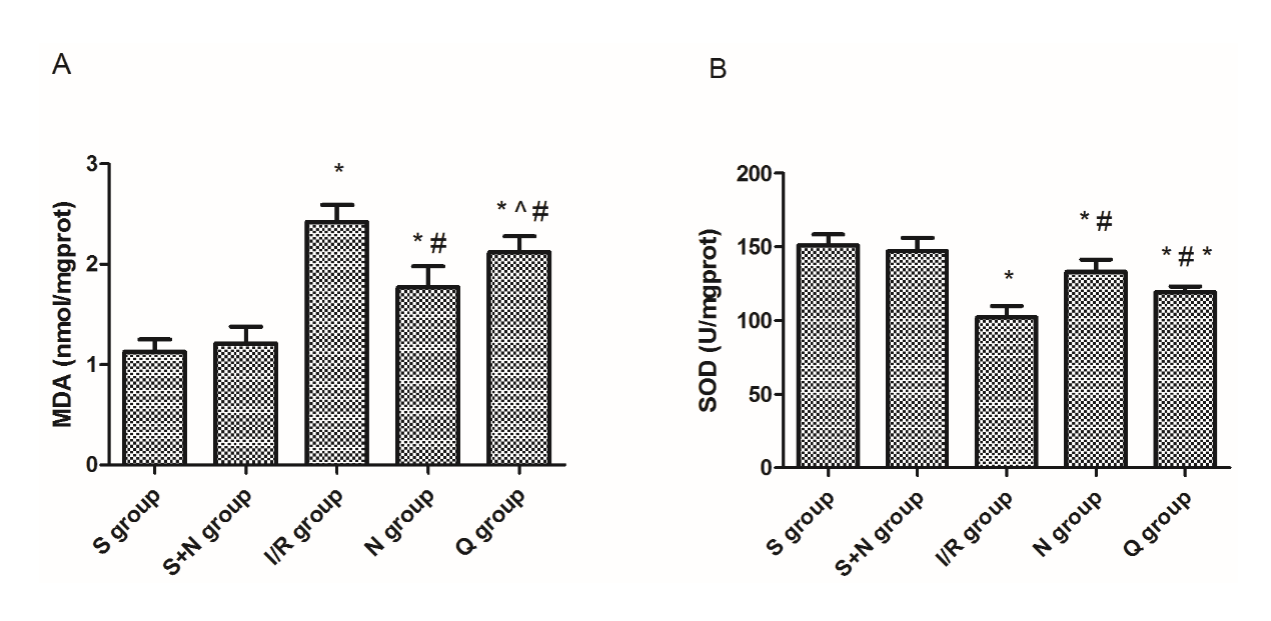
(A) MDA content and (B) SOD activity in five groups at 6 hr post-reperfusion. *P<0.05 vs the sham group, #*P*<0.05 vs the I/R group, ^*P*<0.05 vs the NaHS group. S, sham group; S+N, sham + NaHS group; I/R, ischemia-reperfusion group; N, NaHS (sodium hydrosulfide) group; Q, quercetin + NaHS group

**Figure 6:**
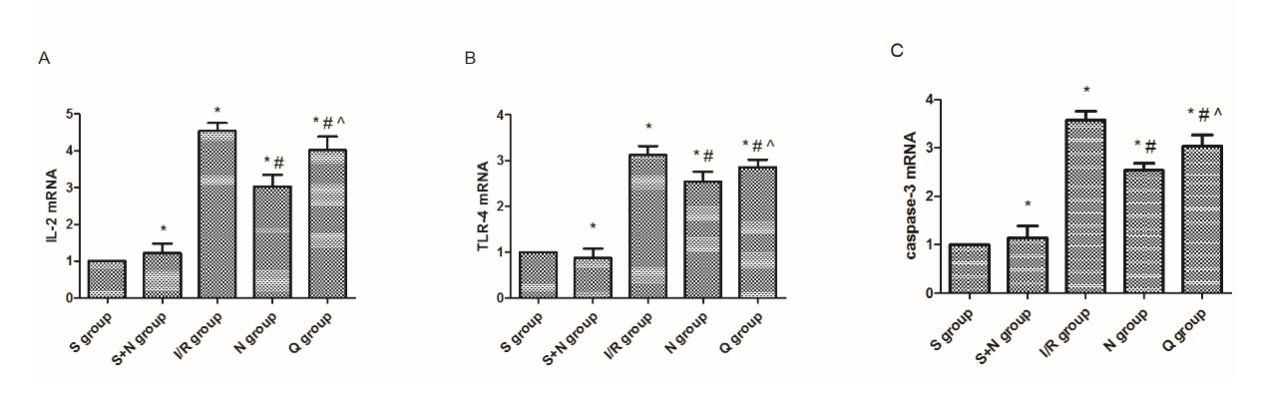
mRNA levels of (A) IL-2, (B) TLR-4, and (C) caspase-3 in five groups at 6 hr post-reperfusion. *P<0.05 vs the sham group, #*P*<0.05 vs the I/R group, ^*P*<0.05 vs the NaHS group. S, sham group; S+N, sham + NaHS group; I/R, ischemia-reperfusion group; N, NaHS (sodium hydrosulfide) group; Q, quercetin + NaHS group

**Figure 7:**
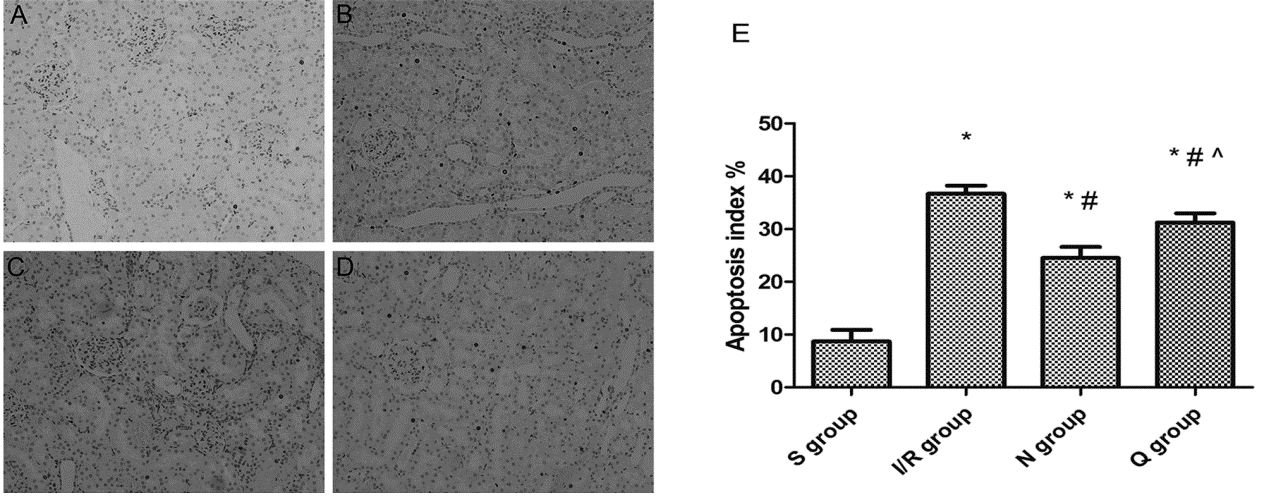
TUNEL staining. Renal apoptosis in the five groups at 6 hr post-reperfusion (magnification, ×200). (A) Sham group, (B) Ischemia-reperfusion group, (C) NaHS group and (D) quercetin +NaHS group. NaHS, sodium hydrosulfide; TUNEL, terminal deoxynucleotidyl transferase dUTP nick and labeling. *P<0.05 vs the sham group, #*P*<0.05 vs the I/R group, ^*P*<0.05 vs the NaHS group. S, sham group; S+N, sham + NaHS group; I/R, ischemia-reperfusion group; N, NaHS (sodium hydrosulfide) group; Q, quercetin + NaHS group

***Immunohistochemistry***

The expression of HSP 27, HO-1, and HSP 70 was analyzed by immunohistochemistry staining. The staining was performed by using goat antibodies against rat HSP 27 (sc-1048; Santa Cruz Biotechnology, Santa Cruz, CA), HSP 70 (sc-1060; Santa Cruz Biotechnology, Santa Cruz, CA) and rabbit antibodies against rat (sc-10789; Santa Cruz Biotechnology, Santa Cruz, CA). All steps were performed following the manufacturer’s instructions (Thermo Fisher Scientific, Waltham, MA, USA) and the results were evaluated by comparing the staining intensity using microscopic examination.

***Western blot analysis***

All renal tissue proteins were extracted and quantified via the bicinchoninic acid assay (BCA). Briefly, equivalent weights of protein samples (40 μg/lane) were separated on 10% sodium dodecyl sulfate-polyacrylamide gel electrophoresis (SDS-PAGE) and then transferred to a nitrocellulose membrane. After that, the membrane was blocked by 5% non-fat milk in Tris-buffered saline and Tween 20 buffer, then incubated with primary antibody against NF-kB (p65; sc-8008, Santa Cruz Biotechnology, Santa Cruz, CA), HSP 27, HO-1, and HSP 70 at 4 ^°^C overnight. After being extensively washed with TBST buffer, such membranes were incubated with secondary antibodies for 1 hr. All specific bands were visualized by an ECL system kit (Pierce Biotechnology, Beijing, China). Optical densities were detected using ImageJ software (NIH, Bethesda, MD, USA).

***Reverse transcription-quantitative polymerase chain reaction (RT-qPCR)***

Total RNA was extracted from equivalent weights of 100 mg each renal tissue sample using TRIzol reagent (Invitrogen Life Technologies, Carlsbad, CA, USA) and the RNA purity was detected by spectrophotometry using a 722-ultraviolet spectrophotometer (JKI Co, Ltd, Shanghai, China) at a score of 1.8–2.0 from the optical density (OD) ratio of 260/280 nm. Then first-strand cDNA was synthesized by using random primers and M-MLV Reverse Transcriptase (Promega Corporation, WI, USA). Subsequently, the cDNA was amplified by qPCR via an Applied Biosystems SYBR Green mix kit (ABI, CA, USA) and the ABI 7900 Real-Time PCR system (ABI, CA, USA) according to manufacturer’s instructions. The qPCR was performed as follows: denaturation at 94 ^°^C for 4 min, 40 cycles of amplification at 94 ^°^C for 30 sec, then hybridization at 56 ^°^C for 30 sec and extension at 72 ^°^C for 30 sec. The quantitation of mRNA was automatically finished using SDS v1.3 software (ABI, CA) and the 2^-∆∆Ct ^method was employed to calculate mRNA expression (12). The primers for IL-2, caspase-3, and TLR4 are listed in Table 1.

***Statistical analysis***

All data are expressed as mean ± standard deviation. Statistical analyses were executed using SPSS 17.0 (SPSS Inc, Chicago, IL, USA). Comparison of parameters among different groups was conducted using one-way analysis and Student-Newman-Keuls test.

## Results

***Hydrogen sulfide treatment increases the expression of HSPs***

The expression of three kinds of HSPs was evaluated by immunohistochemical staining (Figures 1 A-L) and Western blotting (Figures 2 A-D). As we observed, IR induced the expression of HSP 27, HO-1, and HSP 70 both on mRNA and protein level, while H_2_S elevated the expression levels of HSPs even more. In contrast, Quercetin attenuated the expression of HSP 27, HO-1, and HSP 70 induced by H_2_S and IR.

***Induction of HSPs involved in the attenuation of renal IRI by hydrogen sulfide***

To evaluate the impairment of renal function, we observed the changes in renal pathology via microscopy (Figure 3) and measured creatinine and BUN in serum (Figure 4). We found that renal IR led to significant changes in pathology and morphology, as evidenced in tubular dilatation and congestion, medullary hemorrhage, cellular edema, and necrosis in tubular cells. We also found that the serum creatinine (89±6.2 vs 27±3.1 µmol/l, *P*<0.05) and BUN (12.7±1.4 vs 6.7±1.1 mmol/l, *P*<0.05) levels increased at the same time. H_2_S treatment attenuated the pathological changes in kidneys and decreased the serum creatinine (64±5.6 vs 89±6.2 µmol/l, *P*<0.05) and BUN (9.4±1.2 vs 12.7±1.4 mmol/l, *P*<0.05) levels, while quercetin inhibited the renal protective effects of H_2_S significantly, on the other hand. 

***Induction of HSPs downregulated MDA level and elevated SOD activity***

The MDA level and SOD activity were determined to evaluate the levels of lipid peroxidation in renal IR. As we observed, the MDA content increased significantly after 6 hr of reperfusion (2.42±0.17 vs 1.13±0.24 nmol/mg prot, *P*<0.05), while the SOD activity decreased significantly at the same time (112±7 vs 151±8 U/mg prot, *P*<0.05). H_2_S attenuated above changes obviously (MDA 1.57±0.51 vs 2.42±0.17 nmol/mg prot, *P*<0.05; SOD 133±8 vs 112±7 U/mg prot, *P*<0.05), whereas quercetin restrained this attenuation (MDA 2.32±0.21 vs 1.57±0.51 nmol/mg prot, *P*<0.05; SOD 119±4 vs 133±8 U/mg prot, *P*<0.05) (Figure 5).

***Induction of HSPs inhibited inflammation in renal IR***

The expression of NF-kB (p65) (Figure 2 E), IL-2 and TLR-4 (Figures 6 A, B) was measured by Western blotting or RT-qPCR. Results from Western blotting revealed that the expression level of NF-kB was much greater in the IR group compared to the sham group. However, treatment with H_2_S reduced the expression obviously. The relative mRNA expression levels of IL-2 and TLR-4 were detected through RT-qPCR and we get similar results as those found for NF-kB, the mRNA levels of IL-2 and TLR-4 were significantly upregulated (IL-2 1.12±0.12 vs 0.22±0.04, *P*<0.05; TLR-4 1.13±0.14 vs 0.36±0.08, *P*<0.05) in the IR group compared with the sham group and were distinctively reduced (IL-2 0.50±0.05 vs 1.12±0.12, *P*<0.05; TLR-4 0.61±0.11 vs 1.13±0.14, *P*<0.05) in the H_2_S group.

***Induction of HSPs suppressed apoptosis of renal tubular epithelial cells by hydrogen sulfide***

We assessed the expression of caspase-3 mRNA (Figure 6 C) by RT-qPCR and evaluated the AI by TUNEL assays (Figure 7). The results showed that the expressions of caspase-3 mRNA (0.95±0.20 vs 0.14±0.11, *P*<0.05) and AI (36.72±1.54 vs 8.67±2.23, *P*<0.05) in the IR group were much higher than in the sham group, and the TUNEL-positive renal tubular epithelial cells also increased obviously in the IR group compared to the sham group. However, in the H_2_S group, the expression levels of caspase-3 mRNA (0.69±0.03 vs 0.95±0.20, *P*<0.05) and AI (24.51±2.10 vs 36.72±1.54, *P*<0.05) were much lower than in the IR group, so was the number of TUNEL-positive renal tubular epithelial cells.

## Discussion

HSPs are a family of proteins that are produced in response to stressful conditions by cells (13). It has been confirmed that HSPs possess a wide range of biological functions related to the protective effects in cells, and the relationship between HSPs and organ IR also has been reported in previous studies (14-16). In our study, the expression of the three kinds of HSPs increased in different degree after 6 hr of reperfusion, which may be ascribed to a systematic reaction to IR injury. These results were consistent with Zhang’s study (7). In that study, they reported an increased expression of 21 genes in rat kidneys followed early IRI by using gene microarray, and the expression of HSP 70 (43-fold), HO-1(10-fold), and HSP 27 (12-fold) increased most significantly. Hydrogen sulfide (H_2_S), has been identified as an important gasotransmitter in many physiological processes, and lots of studies indicated the protective effect of H_2_S in various forms of organ IRI, including renal IRI (17-18). However, the mechanism of its protective effect against IRI remains unclear and the relationship between H_2_S and HSPs is still limited. Quercetin, as an inhibitor of HSPs, can suppress the expression of these proteins by interfering with their transcription (19). In our present study, we injected quercetin (100 mg/kg) intraperitoneally 1 hr prior to ischemia as Guo described in a previous study (8). As the data showed, the amount of HSPs increased much more in the NaHS group than in the IR group, whereas such changes were reversed after the use of quercetin. Quercetin partially blocked the expression of HSPs induced by NaHS or IR injury. However, the amount of HSPs in the Quercetin + H_2_S group was still more than that in the IR group. That is to say, H_2_S directly induced higher expression of HSPs than IR injury and such high expression of HSPs resulted in different changes of phenotypes in subsequent studies.

HSPs have been reported to alleviate apoptosis via interfering with caspase activation. In another research, exhaustion of HSP 70 triggered cellular apoptosis by activating caspase-3 *in vitro* (19). HSP 27 and HSP 70 can both inhibit apoptosis upstream and downstream of mitochondria, interfere with apoptosome formation and caspase activation (20). Furthermore, HSP 70 can protect cells against oxidative stress. HSP 70 involves in the process of anti-oxidation by inhibition of lipid peroxidation, enhancement of protective SOD activity, and repair of proximal tubule structure after ischemia (21). 

HO-1 (HSP32) is a bona fide stress protein that can be induced in various cell types, including epithelial, endothelial, smooth muscle cells, and others. It has been studied widely in organ transplantation realm for its anti-apoptotic, anti-inflammatory, and anti-proliferation actions (22-24). HO-1 and its product of degradation have been associated with a decreased infiltration of granulocytes, downregulation of inflammatory factors like TLR-4, IL-1, IL-2 et, reduction of oxygen radicals, regulation of immune activation, and other protective effects against IR injury (25, 26).

Previous studies have shown that overexpression of HSP 27 usually induces a decrease of reactive oxygen species (ROS) and suppresses the release of pro-inflammatory factors. HSP 27 can also block cytochrome c-induced caspase activation and influence cellular apoptosis through regulating the ubiquitination or degradation of some proteins like p27kip1 in response to stress factors. It has been reported that HSP 27 involves in providing protection and stabilization to the cytoskeleton in an unstressed cell state by inhibition of the disassociation of microfibrils and actin. So, this protein may be of great significance in maintaining the integrity of the epithelium and endothelium to various stresses (27). 

ROS are important factors in the development of organ IR injury due to their direct damage to proteins and membranes and indirect damage by acting pro-apoptotic pathways (28). Normally, SOD level reflects the efficiency of scavenging oxygen free radicals in the body, and MDA level is used to assess the extent of cell injury under oxidative stress (29). As our present study showed, SOD levels increased and MDA levels decreased after the treatment of H_2_S. However, the results were reversed after the use of quercetin. Taken together, these results suggested that H_2_S induced the expression of HSPs and ameliorated renal IR injury caused by lipid oxidation.

NF-κB is a kind of nuclear transcription factor with great importance in regulating the expression of a number of genes associated with the regulation of inflammation, apoptosis, and tumorigenesis (30). Previous studies have shown that multiple stressors including IR can induce the release of NF-κB p65-p50 dimer and degradation of IκB so as to activate the NF-κB signaling pathway (31). The current study has demonstrated that H_2_S induced the expression of HSPs and further inhibited the expression level of NF-κB, while the results were reversed under the presence of quercetin.

## Conclusion

Our present study indicated that the administration of H_2_S attenuated lipid oxidation, inhibited inflammatory response, and cellular apoptosis, such protective effects against renal IR injury may be ascribed to the induction of HSPs.

## Conflicts of Interest

All authors declare no conflicts of interest.
